# The Hospital Survey and Its Lessons

**Published:** 1920-07-10

**Authors:** 


### The Hospital

The Workers' Newspaper of Administrative Medicine and Institutional Life, Administration, National Insurance and Health.

No. 1779, Vol. LXVIII. SATURDAY, JULY 10, 1920. PRICE TWOPENCE

### THE HOSPITAL SURVEY AND ITS LESSONS.

Sir Napier Burnett truly says, in his notes on the extended survey of the hospitals of the Kingdom, that up to the year 1914 there was no hospital problem from the point of view of finance. It was true of that year, as it may be said of this, that no fully adequate hospital system was to be found. There were waiting-lists, there were improvements needed in the special departments for diagnosis and treatment, much more money could have been usefully expended, but within the limits set by the generosity of the public the hospitals were not in debt, and, indeed, had a reserve which lasted them probably to the end of 1918, when, considered as a whole, as the survey indicates, the voluntary hospitals were in a satisfactory financial condition.

It says much of the affection with which the voluntary system is regarded by the public that it should have successfully weathered the storm so long. It was only when everybody was war-weary and harassed by taxation and high prices that the fabric, in this one matter of money and in nothing else, began to crumble. Even then the volume of charity did not diminish, but, left entirely to spontaneous benevolence, no longer continued to increase. It was then that the guiding hand of such bodies as the Red Cross Society and our Metropolitan Central Funds would have been most efficacious, by throwing in the weight of their influence and their power of organisation. But the Red Cross Society was engaged elsewhere, and the Central Funds had not awakened to the necessity of action. Now that they have thrown their whole weight into the campaign we hope and we believe that the worst point has been reached and passed. But even with this said, a source of supreme pride is the revelation of the survey that during the five years ending 1919 the income of the provincial hospitals (as could probably also be said of the London hospitals) exceeded the expenditure. Not by much, it must be noted, and always bearing in mind the fact that a large mass of postponed repairs and improvements represented a cumulated debt of no mean proportions.

The survey, for all to read with the attention that its great importance demands, is to be found upon another page. It is our present duty to deal with the lessons it inculcates. Those lessons are generally summarised by Sir Arthur Stanley in the covering letter that has been prefaced to the survey. To start comfortably, to start with a clean slate, it is, in Sir Arthur's opinion, necessary to wipe off the war debts of the hospitals, debts which are represented by bank loans and deferred repairs to the fabrics of the institutions. This, he hopes, may be done by voluntary effort, but failing that a call upon the Exchequer may be necessary—a claim which is just because it refers directly to liability caused during, and largely on account of, the war.

So much for what must be regarded financially as a matter of capital. When we come to income for maintenance it is with a sense of no small pleasure that we recognise that what is proposed by Sir Arthur Stanley, who must be regarded as one of the chief leaders of hospital thought in this country, is what has been time after time advocated by this journal, representing as it can fairly claim to do a reflection of the collective opinions of hospital workers, medical and lay, honorary and stipendiary. Contributions towards cost of maintenance, scientifically assessed under the Almoner system, from patients in accordance with their circumstance, rightly takes a foremost place in the proposed programme. The organisation of systematic collections from workpeople and employers of labour, in respect of which we have always, with great confidence as to its productive power, advanced not merely general recommendations but carefully-worked-out schemes of campaign, is another plank in the Red Cross platform.

But income is not the only consideration. Unlimited income can, indeed, be harmful, as we have learned too well during the war. Careful expenditure has an equal bearing on a financial problem when we come to balance the accounts. Expenditure, we mean, which is not merely careful but economical in the best and widest sense, expenditure of money and the forces that money can produce to the best advantage—not lavishly but wisely, with the intention of producing the exact effect required. Expenditure such as this plainly cannot be carried on without the help of a competent authority to examine hospital accounts such as Sir Arthur Stanley suggests. We have always extended this suggestion by the proposal that accounts should be audited as well as examined by a competent authority or by licensed auditors acting under such an authority. But that may come in good time. What is needed at present is the critical examination of accounts by a central body, and one, as experience has taught us is necessary, with the power of the purse behind it.

Sir Arthur Stanley suggests the consolidated appeal. This is a method of increasing support which we have already mentioned, and shall deal with later at greater length. It is a matter which bristles with difficulties, and is in danger of infringing upon the domain of those whose energy has placed them in the sound position not attained by less vigorous people. It is a question which requires the most careful consideration, and cannot with any advantage be fully discussed at the moment.

Another matter referred to is the closer co-operation with the Poor-Law infirmaries, in which there are a large number of vacant beds that might be utilised to relieve the pressure on existing voluntary hospitals. If we mistake not, this is a point which will find a prominent place in the general health scheme under legislation now being prepared by the Ministry of Health. It is evidenced from all sides that such co-operation is possible and desirable.

Sir Napier Burnett's survey and Sir Arthur Stanley's covering remarks reveal the vitality of the voluntary system in a striking manner; they will do much to hearten those who are fighting against strong odds, and having cleared the ground will enable the Red Cross Society to proceed with their plucky effort to relieve the provincial hospitals of that part of their burden that is pressing too heavily upon them.

